# Clinical Holistic Medicine: The Patient with Multiple Diseases

**DOI:** 10.1100/tsw.2005.42

**Published:** 2005-04-12

**Authors:** Søren Ventegodt, Joav Merrick

**Affiliations:** ^1^ Nordic School of Holistic Medicine and Quality of Life Research Center, Teglgårdstræde 4-8, DK-1452 Copenhagen K, Denmark; ^2^ Institute of Child Health and Human Development, Faculty of Health Sciences, Ben Gurion University of the Negev, Beer-Sheva, Office of the Medical Director, Division for Mental Retardation, Ministry of Social Affairs, Jerusalem, Israel

**Keywords:** quality of life, QOL, philosophy, human development, holistic medicine, public health, holistic health, holistic process theory, life mission theory, group therapy, tinnitus, multidisease syndrome, Denmark

## Abstract

In clinical practice, patients can present with many different diseases, often both somatic and mental. Holistic medicine will try to see the diseases as a whole, as symptoms of a more fundamental imbalance in the state of being. The holistic physician must help the patient to recover existence and a good relationship with self. According to the life mission theory, theory of character, and holistic process theory of healing, recovering the purpose of life (the life mission) is essential for the patient to regain life, love, and trust in order to find happiness and realize the true purpose of life. We illustrate the power of the holistic medical approach with a case study of an invalidated female artist, aged 42 years, who suffered from multiple severe health problems, many of which had been chronic for years. She had a combination of neurological disturbances (tinnitus, migraine, minor hallucinations), immunological disturbances (recurrent herpes simplex, phlegm in the throat, fungal infection in the crotch), hormonal disturbances (14 days of menstruation in each cycle), muscle disturbances (neck tensions), mental disturbances (tendency to cry, inferiority feeling, mild depression, desolation, anxiety), abdominal complaints, hemorrhoids, and more. The treatment was a combined strategy of improving the general quality of life, recovering her human character and purpose of life (“renewing the patients life energy”, “balancing her global information system”), and processing the local blockages, thus healing most of her many different diseases in a treatment using 30 h of intense holistic therapy over a period of 18 months.

## INTRODUCTION

From the biomedical perspective, a disease is a well-defined entity that can be understood in its own right and treated independently of the person and almost independently of other diseases and conditions in this person. A physical disease (for example, an infection) can be treated independently of a mental disease (for example, depression) naturally taking into consideration the interactive effect of any drugs. There has been very little research on how to treat the patient with many diseases (a recent search on www.pubmed.gov did not reveal any controlled clinical studies with such patients). From the holistic medical perspective, diseases, if not caused by genes or by traumatic impacts from the outside (like asbestos, oncogenic tar from smoking, etc.), are caused by lack of coherence[1,2] due to inner conflicts that disturb the fine order of the organism and its cells[3,4,5,6,7,8,9,10].

When a patient, such as the one we are going to present in this paper, has a combination of neurological disturbances (tinnitus, migraine with severe scotomata, minor hallucinations), immunological disturbances (recurrent herpes simplex 1 and 2 on lip and labia, phlegm in the throat, fungal infection), hormonal disturbances (14 days of menstruation in each cycle), muscle disturbances (neck tensions), psychic disturbances (tendency to cry, inferiority feeling, mild depression, feelings of desolation, anxiety), abdominal complaints, hemorrhoids, and more, the approach of a biomedically trained physician would be to start from an end, treating what is possible and leaving the rest untreated. When the patient has a moderate number of diseases, this strategy often works wonders, but when she has 20 problems, it mostly will not work. Biomedicine does not seem effective with this group of patients.

From a holistic point of view, all the organ systems are intimately connected and they share the same information system[3,4,5,6,7,8,9,10,11,12,13,14,15,16] or they share the same “life energy”. So, the many diseases are a function of the person being low on energy or being disturbed *informationally*. The cause is due to fundamental inner conflicts that bind the energy in blockages that are held by the different tissues and these are revealed by the various symptoms. Interestingly, there are two kinds of causal patterns in holistic medicine: a positive pattern of a blockage giving a local disease and a negative pattern of the body and organism being drained of its energy. The multidisease syndrome is often caused by a combination of the two. Tinnitus is a typical manifestation of the negative pattern, as is severe chronic pain in the bladder and kidney. Cancer is a typical manifestation of the positive pattern. When it comes to the holistic treatment of the diseases, a negative pattern must be handled in a long, systematic process of rebuilding the person as her true self. A positive pattern is often easier and faster to treat as the whole existence is less affected. Whatever the chosen course of the treatment, it always starts with the physician’s love or intense care and goodness for his patient, inviting the patient’s trust and allowing the physician to hold and cure[17].

## CLINICAL HOLISTIC MEDICINE

The life mission theory[18,19,20,21,22,23] states that everybody has a purpose of life or huge talent. Happiness comes from living this purpose and succeeding in expressing the core talent in your life. To do this, it is important to develop as a person into what is known as the natural condition, a condition where the person knows himself and uses all his efforts to achieve what is most important for him. The holistic process theory of healing[17,24,25,26] and the related quality of life theories[11,12,13] state that the return to the natural state of being is possible whenever the person gets the resources needed for existential healing. The resources needed are “holding” in the dimensions of awareness, respect, care, acknowledgment, and acceptance with support and processing in the dimensions of feeling, understanding, and letting go of negative attitudes and beliefs. The preconditions for holistic healing to take place are trust and the intention for the healing to take place. Existential healing is not a local healing of any tissue, but a healing of the wholeness of the person, making him much more resourceful, loving, and knowledgeable of himself, his own needs, and his wishes. In letting go of negative attitudes and beliefs, the person returns to a more responsible existential position and an improved quality of life. The philosophical change of the person healing is often a change towards preferring difficult problems and challenges instead of avoiding difficulties in life[3,4,5,6,7,8,9,10]. The person who becomes happier and more resourceful often also becomes more healthy, more talented, and more able to function[27,28,29].

## A CASE STUDY ON MULTIPLE DISEASE

When we began our professional career in general practice, we soon discovered that it was fairly safe to ask the patient the relevant question: "What can *you* do to get better?" This simple question would often make the patient become distant, silent, and pensive and so an important process had begun. In the case of the following patient, a 42-year-old female artist whom we will call Mia, we found that additional measures were required. Following 10 years of illness in the form of various ailments ranging from migraine and herpes simplex 1 and 2 to treatment-resistant genital warts and depression, she had come to accept that there was no solution to her problems and merely wanted the standard medication for her symptoms. However, she had a problem that worried her most — her tinnitus had become worse, much worse in fact, and would it be at all possible to do something about it?

Despite her age, Mia was in very poor condition physically and mentally, but she possessed something special: alertness and an interest in the spiritual world. She wanted to develop as a person and that meant that she was ready to assume responsibility and take the rather bitter, holistic medicine that the clinic could offer her. Not once during the period when she came to the clinic, which lasted over a year, was she prescribed a single pill. She met the therapist (SV) in a good and sincere way and after her first appointment, she went home with four exercises that she was very keen on doing for her next appointment 14 days later. Exactly 14 days later, she arrived with ten closely written A4 pages. She surprised the therapist because she provided complete solutions to all tasks, which she had taken very seriously and worked on with great determination. Clearly, she regarded the treatment as a real opportunity to recover, primarily from her debilitating tinnitus. She understood the working methods of the holistic clinic, believed in them, and was very motivated.

Processing her painful personal history took her directly to her life purpose. Following this acknowledgment, her art began to flourish and grow like never before. Suddenly, she could do things that she had not been able to do before and her art expressed her new state of acceptance and understanding of good and evil, beautiful and ugly, muck and mire, and sky and light. Occasionally, the therapist appeared as a counselor in Mia's dreams. At some point, she revealed that she was in love with the therapist, which is common when a patient experiences genuine help. Fortunately, it will pass away, but it can be quite a strain at the time. When she acknowledged her life purpose, Mia became able to manage on her own and able to develop further without our help. The therapeutic work of guiding her through the pain that made her ill and blocked her enjoyment of life and self-expression is finished. Her body and soul have largely healed, her tinnitus is almost gone, and she cannot hear it at all most of the time. Obviously, this patient may become physically ill again, but her resistance and inner equilibrium appear to be much greater than before, so next time she is likely to recover much faster.

It is worth noticing how the pain shifts from body to mind and existence in the course of the conversations. By the 12th conversation, she was on the verge of becoming psychotic. By the 18th conversation, after about 1 year of therapy, the patient no longer had any physical complaints, but still struggled with difficult existential problems. We talked about the world being "evil" and she was coached to see the world as both good and evil. Taking responsibility for our lives means that we can relate to both good and bad things in the world in a constructive and clear way. Following a total of 23 conversations over a period of 18 months, she gained control of all her talents, became happy, and is functioning well with a new job. Below are the chart abstracts of the 23 conversations.

> **Female, aged 42 years, with tinnitus, migraine, herpes simplex 1 and 2, lower back pain, treatment-resistant genital warts, sun allergy, depression, etc.:** First quality-of-life (QOL) conversation: Presents with multiple problems: Menstruate 14 days in each cycle, suffers from migraine with severe scotomata blinding her for up to 20 minutes, herpes simplex 1 and 2 on lip and on labium minus and labium majus, both on left side, neck tensions, phlegm in the throat, tendency to cry, inferiority feeling, fungal infection in the crotch, haemorrhoids and severe abdominal complaints previously but not at present, tinnitus, tendency towards depression, occasional minor hallucinations in the form of symbols, signs and aura vision which she cannot control, feelings of desolation, anxiety….. Spent one year in a foster home as an infant, then adopted. Present either in her head/rational mind or in her body and sexuality or in her mind and the spiritual dimension. By contrast, she is rarely centred in her heart/soul, although she considers herself an emotional person. Social: Has had a boyfriend for one year, whom she has known for 17 years. Has recently abandoned her lover. On examination: neither an acute nor a chronic affliction, not psychotic, but skilled at escaping from herself and from emotional pain. All symptoms might have the same cause. EXERCISE: the patient is instructed to search for such a cause. In addition, she will compile a complete list of all symptoms and their causes, as far as possible.

Second QOL conversation: Arrives with about ten written pages – well done. She has sorted out all her symptoms and feels that it is very much about her not being allowed to be the woman she is. She feels as though something is missing in her history, so the next exercise is to go home and write down her relevant history. Since many of her problems have occurred in connection with a relationship, she will focus on that. She has realised that all her problems relate to “suffering in her senses”: eyes, ears, mouth, neck, skin and reproductive organs. Next appointment in two weeks. Patient wrote concerning EXERCISE:1. Suggested common cause: entire organism craving attention by displaying symptoms. There appears to be a need for care and love. 2. List of all problems:

MIGRAINE: Onset at age 19 years, new relationship with C. Lived at home. Parents worried about his lack of initiative to make a living. Snowing, leaking water pipes. I am washing and tidying ... suddenly I cannot see ... later, frequent occurrence at times of stress and menstruation, neck tension.

HERPES SIMPLEX 1 and 2: 27 years old, new relationship with E. Ended relationship with J. Hospitalised after trip to Africa with various diseases, amoebic dysentery and malaria. Later, sometimes in stressful situations, monthly after menstruation.

TENSION IN NECK AND HEAD: Onset at age 16-17, relationship with A. Parents opposed it. He is violent. Aggravated at end of schooling and start of higher education. Later at times of stress and insecurity.

PHLEGM IN THROAT: 40 years old, following throat inflammation. Alone and recently divorced in small town. New relationship with O on and off.

BLACK SPOTS IN VISION: 28 years old, after Africa, moved to Norway, new relationship with E.

TINNITUS: 41 years old, new relationship, new home, new job, everything new, new life. Inflammation, ears, loud noises at work.

LONG MENSTRUATIONS: 39 years old (kundalini rising with Qi-gong, in love with new spiritual man, weight loss, sleeplessness, diarrhoea).

LOW BACK PAIN: In connection with hard work and lifting.

FUNGAL INFECTION IN THE VAGINA: 27 years old, new relationship with E ... after Africa and hospitalisation, gardnerella... again following experimental sex and penicillin.

GENITAL WARTS: 39 years old, in love again without sex, in old relationship (transmitted from him?).

> CHILDLESSNESS: 27 years old, new relationship, no contraception. Complaints following abortion at age 22 or inter-uterine device. Deformity!
>
> URGE TO VOID: When nervous, in new places.
>
> PIGMENTARY DISTURBANCE: 35 years old, therapy, search for biological parents.
>
> SUN ALLERGY: 13 years old, playing in the early spring sunshine. Now, severe again. Since then at sunstroke on Crete at age 20.
>
> SLEEPING PROBLEMS: Baby, did not sleep with biological mother at nights because of colic. At age 22, too much work, from old relationship to a new one, medication (anxiety neurosis – medication prescribed by doctor, sleeping pills, side-effects: insomnia). Later on when I have to get up early or miss new places.
>
> MINOR DEPRESSIVE EPISODES: 22 years old, nervous breakdown after six months of insomnia. Following manic episodes, or if I am on my own for too long and no one calls me.
>
> PARANOIA: Puberty, after having been caught and punished several times for shoplifting. At age 17, reactivated when smoking cannabis. Feeling of being under surveillance, different, not good enough (childhood).
>
> ABSENT-MINDED/FORGETFUL: 30 years old, many different jobs and ambitions, again when busy or emotionally unbalanced.
>
> REJECTED CHILD: Early childhood, if friends could not come out to play, later if friends did not call much. In all relationships from age 22 and onwards, generally when received insufficient attention.
>
> EXPECTATIONS OF PARADISE LONGING: From early childhood, a longing for God. Childhood with material wealth, emotional poverty. At age 36 a revelation, mournful moments at church. At age 40, divorce, new life, weary of life and longing for death, for going home. Longing for more contact with my own essence after having experienced it.
>
> MENTALLY ORIENTED: 19 years old and in steady relationship with routine sex. Often, the sexual activity moves into the head, and I lose contact with my entire body, the sexual desire. Then present in head and crotch.
>
> SELF-FOCUS: Self-absorbed due to ailments.
>
> HIGH ENERGY/COLLAPSE: 22 years old, nervous breakdown due to working overtime and insomnia, later tendency to work more than body and mind can handle, then collapse from exhaustion or depression.
>
> "HYPERSENSITIVITY": From childhood, exposed to anger and hysteria. Later, hypersensitive to other people's attitudes and moods. Sense other people's moods and become affected as if they were my own.
>
> WITHDRAWAL: Childhood, withdrew into myself and my own physical space to have peace and quiet. Later, I need to be alone in my physical space following get-togethers, otherwise I feel invaded.
>
> HEAVY DEMANDS: Childhood, very responsible and helpful. Now I make excessive demands on myself, to be attentive and helpful in relation to other people.
>
> TENSION: Shyness from childhood, reduced self-esteem, feeling of being different. Adult shyness, feels the body become tense because it wants to escape the situation, because it feels insecure.
>
> SHYNESS/OVER-CONFIDENCE: Puberty, developed a tough personality through idolisation. Now I can display over-confidence in tight situations, I become tired afterwards.

This list is a fine example of how we become ill when we simply do not have the vital energy to stay in one piece when we are young. Many patients disclose a similar list when forced to compile a complete list of all symptoms and ailments. Usually, the regular physician sees only the tip of the iceberg and the patient sees only one symptom at a time and does not realize that there may be 20 different symptoms appearing one after the other.

> Third QOL conversation: Good progress in understanding the problems. Patient can now partly control tinnitus herself. There is no love in her life. She does not want to be vulnerable and is therefore unable to give and accept love. She protects herself by being masculine and domineering. EXERCISE: Accept your vulnerability and responsiveness, love the vulnerability of femininity. Take stock of your current symptoms. For next time: Give-and-take flow must be restored.
>
> Fourth QOL conversation: The patient relates that a lot is happening inside her in terms of order and understanding. She believes her ailments derive from the suppression of the vulnerable woman, which she has worked on since last time. Brings full-size picture of herself as vulnerable. Has been angry, had a fit where she smashed a door. Has celebrated her femininity and wearing a dress for the first time. Six out of 13 physical symptoms have improved (migraine, herpes both on lips and genitals, which she usually has for months, did not develop beyond a herpes "tickle" at the sites without atrovir, no longer troubled by "black spots" in her visual field, low back pain and fungal infection in the vagina have improved and genital warts "now look white as though they are about to go away", three of the physical symptoms have become worse (urge to void, tension in head and neck and tinnitus), while the rest are unchanged (phlegm in the throat, protracted menstruation, childlessness and pigmentary disturbance). The six psychological symptoms have improved substantially – easier to fall asleep, not so depressed, no episodes of paranoia, not so forgetful and absent-minded, does not think so much about being the rejected child, and is not "manic". EXERCISE: Enjoy being a woman, be good to yourself, but accept the suffering that sometimes emerge.
>
> Fifth QOL conversation: She is feeling much better – the following have disappeared: migraine, herpes simplex 1 and 2, black spots in the visual field, low back pain, fungal infection in the vagina. Improvement: tension in neck/head, phlegm in the throat, urge to void and pigmentary changes disappearing from her hands, patient says. Psychologically, four of the six symptoms are gone: sleeping problems, depression, feeling of being a rejected child (patient dreamed that she took care of it), she is less forgetful and absent-minded, but her paranoia is unchanged. New symptom: Has a sensation of fullness in the stomach. Patient feels disabled by her childhood and we discuss the nature of the damage. "I have lost contact with myself". EXERCISE for next time: Write one A4 page about each question: Who are you? What are you? Where are you? Why are you? If the patient can handle this difficult exercise, I (SV) think we will make a breakthrough soon.
>
> Sixth QOL conversation: Patient says it is as though a miracle has happened. She has worked on who and what and how she is and brings many closely written pages. She has had some problems with her boyfriend, however, who has moved in with her. She makes sexual demands on him, and he backs off. We talk about her making demands, because she feels that "she has a need" and about the dependency being a manifestation of her escaping from herself instead of being at ease in her own centre. When confronted with this, the patient cries. We talk about being at ease in one's centre and being balanced. EXERCISE: Sit and be who you are. Stop making demands, stop criticising and controlling. Centre physically, in terms of your thoughts, in terms of your emotions.

Seventh QOL conversation: Patient has gone through a long and significant, painful process, dealing with the early loss of both father and mother. She was placed in a foster home. We discuss that. EXERCISE: Write down all the essential events from that time in details. Search for your life-denying decisions. All negative feelings are rooted in a statement (e.g. “I’m going to be sick”, “I feel like dying”) that says it all. Find it.

Eighth QOL conversation: Patient has had great success in touring as an artist and sold nearly everything she has painted. Is happy on arrival. Has established contact with the evil inside her. EXERCISE: Describe in detail all the evil in your life, including the circumstances of your conception (rape).

Ninth QOL conversation: Patient has relived a vast amount of evil since last. Subsequently, she has had a vision, where – perhaps God – showed her as a ray of light in the darkness, whose purpose was to transform the darkness. We talk about her personal mission, which she has formulated first as “I am the light transforming the darkness”, then as “I am light in the darkness.” Then she becomes very sad and ends up curling into a foetal position, in contact with the events where she denied her mission. EXERCISE for next time: Find all the events in your life, when you have denied being a light in the darkness. What did you decide?

Tenth QOL conversation: Patient is very sad on arrival. “I miss my mother,” she says. We work on that statement: “I miss you, Mum,” and the patient cries. She has had three mothers, first a biological mother who gave her up to a foster home, when she was a few months old. Then a mother in the form of the caregiver at the foster home and finally an adoptive mother. Early on she made the following decisions: The man/father does not see me and the woman/mother is going through hell, because of the man. EXERCISE: Let go of these statements, when you have found their exact wording. Finally, the patient has a very severe headache, treated with massage of very tense neck muscles, a hard knot on the right side corresponding to cervical vertebrae C4, C5 and C6.

Eleventh QOL conversation: Severe problems with anxiety and pity for her partner. Is very frightened and wants to sell the flat, which he will not move out of, to get him out. He nearly killed an ex-girlfriend, when she left him, she was hit on the back of the head with a bottle and hospitalised with head injury. She does not want to involve the police, which is what I recommend. EXERCISE: The exercise for next time is to accomplish a respectful and proper farewell and make arrangements for the break-up and for her partner to move out. We discuss her lacking grounding, and the solar plexus and heart being weak and needing attention.

Twelfth QOL conversation: Patient has been moving at a very fast pace and was apparently close to going insane yesterday. Today she is more grounded and possible to talk to. We talk about her feeling a fundamental pain in facing reality, that is, the pain residing in her personal mission: being a light in the darkness. I recommend her to see a private psychiatrist, which she rejects. I give her the following EXERCISE: she has to make a determined effort to become grounded and slow down, otherwise things may easily go wrong. When the emotional pain moves from the body to existence and cannot be accommodated there, it often returns to the mind, which easily becomes overloaded: the patient becomes psychotic. It is important to understand this mechanism to allow the treatment to last for as long as the patient needs and to avoid any unnecessary human tragedies. To prevent psychoses, the physician has to slow down the over-zealous patient, for example by instructing the patient only to work on herself for 30 minutes a day; then, the patient should turn to other activities when she can no longer handle the responsibility for the emotional pain uncovered by the exercises and homework.

Thirteenth QOL conversation: Patient has broken up with her partner and now wants to live in celibacy for two years. I suggest six months. She has produced a fantastic picture of positive and joyous fertilisation. She is working on very early and harsh rejection by her parents, which is healed by her reconnecting to humanity in her art, which now deals with people and bodies. Lying on the couch, she cries and talks about when she was let down. “I gave up,” she says, and I let her enter the sensation of giving up and “dying” on the couch. In my assessment, the patient is making good progress, processing ever deeper and more painful layers of her consciousness. Her tendency to become psychotic has decreased.

Telephone conversation 1: Patient feels well, needs grounding, has dropped her other alternative therapist and is going for Rosen sessions. Wants to go into gestalt therapy. Can return to me in three months or if required.

Telephone conversation 2: Things are going well in the Rosen therapy, working on her relationship to muck – physically and figuratively. Works on the statement, “I don’t want to grow up”. Stopped growing at 13 years. Watched Tin Drum and broke down completely because of that statement.

Telephone conversation 3: Very happy about sessions with gestalt therapist, who “has so many colleagues” that help her. “The other day a pixie removed a load of muck from my third eye,” the patient says, and “I feel so happy every day.” “I don’t feel ill anymore – sometimes I hear a tiny noise in my ear, but I know why it’s there, so it goes away.” Apparently, the patient has recovered and is now working on her personal development and will therefore finish the gestalt sessions. Can continue for as long as she needs with Rosen therapy. To return to me for check-up/adjustment in about three months. Patient draws a full-size picture of herself, and as she develops herself during therapy the picture transforms from shadow through pain and agony, ‘naked’ and ‘skeletal’ into a vivid, lovely and life-affirming picture, the primal mother, Eve.

Fourteenth QOL conversation: Brings a list of 30 negative statements, which she has to let go of. I help her with the first one. “I don’t receive anything” – let go with roll. Then on the couch, where “heart blockage” is loosened in the spine (T12). We work on a shift in perspective: from false to true self. EXERCISE: Let go of all the statements one by one until they have all been released. Return in a month with the list, organised by chakras.

Fifteenth QOL conversation: Feels very bad, new gestalt emerging: my task is “to balance yin and yang”, I am in this world to create harmony between man and woman. We work on the gestalt with time line therapy ranging from conception to just after her birth, we go through it three times, the patient is first her mother, then her father, then both to begin with and she sees numerous diabolic images, where father and mother rip off each other’s flesh and skin, like classic devil scenarios from medieval painting. Has to stop here, but should continue. Believed the gestalt’s statement to be the second mission in therapy and that is correct. First mending statement, it would seem, where the pain around her becomes internalised. Next appointment ASAP.

Sixteenth QOL conversation: We continue and process the event from conception to birth four times. The patient enters something very dark, first massive catharsis, then it crystallises apparently as an attempt at abortion with a knitting needle through the brain, foetus 2-3 months old. Patient cooperates with her mother, who wants her dead and commits suicide in terms of energy, constantly sets aside her own life to become quiet, easy and tractable and not difficult or demanding when she was born. Patient sees herself as grey and dead at birth. Process to be continued at another time; the most emotional parts appear to have been removed from the event, but still no absolute clarity. If the patient knows her life purpose, formulated at conception according to the purpose of life theory, it is possible to review the patient's entire life from conception to the present by means of time line therapy. It often promotes a patient's sense of responsibility for his or her own life when all life events are seen in the right context, based on the purpose of being here, which is the meaning of life.

Seventeenth QOL conversation: What is responsibility: "That is when you act in relation to your goal," the patient suggests. I suggest that it is about being a causal soul in relation to your world and enduring the suffering by fighting for the development of your determination. "I am my body," the patient says - long talk about the difference between having and being. Has begun working with great discipline for eight hours a day, which is new to her, feels like an isolated bubble, which I consider a step in the right direction. Patient makes a prayer to God to be allowed to reconnect. We talk about divine creativity; talking about it, she does not seem to fully understand it. EXERCISE: Cosmological upgrading: describe your cosmology based on you as a responsible, causal soul (you are the cause). EXERCISE: Let your pictures make a real difference to people's lives. Then they are sure to sell.

Eighteenth QOL conversation: Has no longer any physical complaints, but is still struggling with existential matters. We talk about the world being evil and the patient cries. "Many people are worried about me and believe that it is not good for me to see you," she says. Who, I ask, and the patient finds it difficult to come up with any names. "Does anyone mind you letting go of your negative perception of the world as an evil place," I ask her, and the patient cannot think of anyone. We work on the statement "the world is evil", and I coach her to see the world as both evil and good (the divine perspective). At first, the patient is unable to say it out loud – "I am breaking a vow of silence to lots and lots of creatures in Heaven, I am going insane," she says. "You are already crazy, there is no need to be scared," I say. We talk about letting go of her standpoints, so that she can sink into life and let "life carry her" instead of her carrying life. Cries and moans, lets go of the evil world. To return in two weeks. EXERCISE: Look at everything as 'both-and', not 'either-or'.

Nineteenth QOL conversation: Has done her exercise very well. Patient "tries to trust in me", tries not to be controlled by her emotions, and so on. Strong defences. Does not want to make a plan for her life and that way assume responsibility. Enter your position as a free soul and formulate your wishes concisely and the road to fulfill them. Abandon the strategy of floating in a stream of emotions. It will take you nowhere. Hardly any progress since her healing session. Life and death are the same, she says, when I point out to her that she may be heading for her own death. Patient takes no responsibility. EXERCISE: Make a plan for the next five years of your life, your personal life, working life, social life, etc. Specified goals at 3 months, 6 months, 12 months, 24 months, 36 months, etc.

Twentieth QOL conversation: Has made a fine plan as agreed for her working life, personal and social life. The very first day after her last appointment she started to feel better, and today she is not suffering at all. She is aware that she is still like a girl in puberty, and we talk about being an adult – and about the significance of becoming ‘re-parented’ in therapy and finding one’s bearings as an adult. Has had breathing difficulties. “Would you like to call your mother and say goodbye?” I ask her. “Uh, don’t say that – fear of death?!” Yes, I say. And press my hands against her chest. Hold your breath. She holds her breath and starts to cry. “Here is the black hole I don’t want to go into anymore,” she says. “But now you are in the soul perspective, and now you can let go of your negative decisions.” “Alright then.” “I want to die,” she repeats, going back in time to when she was about to be conceived and ended up dying as a free soul and becoming the soul she has been ever since. As she says “I want to die”, it is as though she vanishes in a haze, and I have to slap her face a few times to make her return. She becomes satanically angry and I ask her whether she would prefer exorcism. She repeats it and lets go and ends up having a smiling mouth on her chest, where there used to be oppressive fear of death. EXERCISE: Let go of your statements. First repeat it and understand where it comes from. Do not let go until you have acknowledged it clearly. Those you cannot handle, you can bring to me. Begin with the easiest ones. Promises me copies.

Twenty-first QOL conversation: Patient broke down the other day at the social security office and finally managed to ask for help. She could not bear the thought of having employment found for her, because she is working on her art and herself. Cried incessantly and felt completely powerless. Finally, the good mother in the form of society was there for her. That is understandable considering what she has been through. /Suspected post-traumatic stress disorder/. Medical certificate form 205 – three months. Partially unfit for work. Believes herself that she is able to work for a few hours a day. Arrives with 30 negative statements: The world is evil. I am evil. I don’t receive anything. I become wrong. I am worthless. I am all wrong. EXERCISE: Is to convert all these statement into a positive life philosophy. Stating the opposite is not enough (for example “I am wrong” becomes “I am right”), she has to be genuinely creative.

Twenty-second QOL conversation: Has worked very well with life philosophy – 36 statements about herself have found a healthier replacement. Now has to work on her attitudes to the outside world and later also to life inside herself. She suffers from anxiety/paranoia, for instance when she is in brightly lit rooms with many strangers around her. “I steal”, “Other people hurt me” etc. has to be turned into the opposite. EXERCISE: Three A4 pages about her perception of herself, the outside world and life – the three main elements of life philosophy. Compile a list of all the old statements and write them next to the new and truer ones. She is in existential pain, and we talk about this pain being a basic condition that can only be counterbalanced by the fundamental enjoyment contained in life. PLAN: Gestalt therapy/holotropy can be applied now.

Twenty-third QOL conversation: Has written a book about her process that will be finished soon. We talk about having a narrator in the book explain what happens in each chapter in the present tense. Still has problems with sexuality and femininity – perceives herself as androgynous – “I am yin and yang,” she says. I believe a female therapist can help her, so: PLAN: My gestalt therapist, psychotherapy/holotropy can be applied now – for the male/female/sexuality field. Process: “Aspects of life purpose (the Swiss knife)”. We draw her ‘life purpose flower’ with petals: knowledge, insight, creativity, love, colours and energy, joy, play, craziness and wildness, consciousness, exchange, intelligence, life, including sex, etc. EXERCISE: Discover all the petals in the flower = your talents, write 4-7 lines about each quality.

## COMMENTS

The most important aspects of this case history are the following:

- **Recognition of illness.** The patient was suffering physically from 14 diseases or disorders: Migraine, herpes simplex 1 and 2 (mouth and genitals), tension in neck and head, often phlegm in her throat, black interfering spots in the visual field, tinnitus, protracted and heavy menstruation, frequent low back pain, fungal infection in the vagina almost constantly, treatment-resistant genital warts, annoying urge to void, plus sun allergy, pigmentary disturbance, and involuntary childlessness.
- **Psychologically** she had six problems: sleeping problems, minor episodes of depression, paranoia, tendency to be forgetful and absent-minded, painful feeling of having been rejected as a child, and tendency towards mania.
- **Spiritually** she described: longing for more contact with her own essence.
- **Sexually** she described: mentally oriented (often being inside her head) and varying sexual desire.
- **Emotionally** she described: overwhelming shyness, instability, low stress threshold and tendency towards nervous breakdown/collapse, often after working overtime.
- **Socially** she described: hypersensitive to other people's attitudes and moods, tendency to withdraw from social relations, excessive demands on herself, tension, alternating between shyness and sudden overconfidence. The patient's list of complaints would not be unusually long for a patient 30 years older, but she was far too young to be so troubled. It contained about 20 different issues, all intertwined.
- **Causal explanation.** It turns out that the 20, seemingly independent, ailments all derived from one and the same source deep down in the patient's existence. When this source, invariably old pain, was identified and integrated, all the ailments disappeared. This is genuinely effective medicine. The patient herself had thought about a possible underlying cause and, in that connection, she obtained an important insight: all her physical complaints were somehow related to her skin and the other sensory organs. Her suggestion of a central, common cause was: "The entire organism calls for attention by displaying symptoms. There appears to be a need for care and love". Finally, everything was gathered in an impressive diagram of the interrelations. She went from being unloved, through failing to thrive, to escaping from failing to thrive, and onwards through great (heavenly) expectations to merciless disappointment.
- **Ambition, trust, and persistence.** The patient had to work herself into her problems with her senses and sensitivity by reliving all the often-disappointing and painful relationships in her life. I gave her a new exercise, namely, to write a detailed autobiography focusing on love and sexuality. For patients like her, this exercise may take up 100 closely written pages. The patient also did this exercise in 14 days.
- **Searching for truth.** The patient was asked: "Who am I? What am I? Where am I? Why am I?" The focus was on life purpose, which the patient was asked to find. She took this task seriously and returned two weeks later and said: "It is as though a miracle has happened." These are her answers to the four questions:
    1. Who am I? "I am like the cucumber plant growing in my garden, a strong survivor with difficult growing conditions. I am a soul in the shape of a person, a woman with a physical presence, a name, an age and a history. I contain all opportunities and potentials."
    2. What am I? "A free human being, going back to search for my soul, a self less and less overshadowed by the ego."

3. Where am I? “I am in the working process in my spiritual workshop, sometimes a very lonely place to be in and very self-focused.”
4. Why am I here?” “Because the universe wants me to be on Earth.” “My person is needed in this world, I am here for myself and for others.” “I am here now, conceived through rape, constricted in a corset during my entire creation. Unwanted, rejected, thrown back and forth. Ignored.”

- **Responsibility for existential pain.** We can now uncover the earliest and most painful aspects of her personal history. She was conceived under the most dramatic circumstances, when her mother was raped. She was unwanted and adopted as an infant. She was placed in a foster home and suffered terribly. She encountered the pain both at home on her own and together with me at the clinic. She wrote about it: “Pain, pain. Yes, I see that I am at the centre, back at the centre. I see that I have been there many times before drawn into it by numerous therapists. I see that I must stay there, at the centre of the pain ... I went by train to see the doctor, preoccupied with my pain; a woman stops and wants to help me. It touches me to see how much shows up when I do not escape. The bird in my garden is not afraid of me. God shows me love. I can begin to feel love for other people.”
- **Healing through love and contact with God.** The patient is now ready for the classic exercise called “sitting” (Vipassana): sitting and feeling herself and letting go of the tension in her body, making room for emotions and letting her thoughts be clouds drifting in the wind, while she is witnessing everything and noticing how her body is breathing by itself.

Over the course of the therapy, many things happened in her dreams that she wrote about manically and filled several thick diaries with writings about love and her new contact with God. It turned out that the terrible experience of being conceived through rape contained something very beautiful, namely, that her very life purpose had become to bring goodness into all the evil, to bring light into the darkness, apparently as a result of the rape. The reason for the patient’s recovery was that the inner and outer connection was restored, so that all the cells in her body once more could receive all the information required to be healed. Externally, she has the experience of being reunited with the universe or, subjectively, God. Internally, she was reunited with life or, subjectively, her soul and its purpose for being here. In our view, it is also possible to give a purely biological description: Everything is interrelated, at all levels, information flows freely inside the organism and between the organism and the outside world and all parts of the body and mind returned to their natural and healthy condition.

## DISCUSSION

According to the purpose of life theory, the gestalts with repressed existential pain can be placed either in the body or in the mind and brain. In practice, all patients who are very ill display symptoms from both body and mind. Statistically, there is a close relationship between perceived physical health and perceived mental health. This can be investigated by having patients assess these health factors themselves, for example, in a questionnaire as we did in the Quality of Life Survey. According to the purpose of life theory[20] and theory of character[30], mental illness is a direct consequence of denial of our life purpose, where we destroy our own being, doing, or having. As with mental illness, physical illness appears to be highly sensitive to the restoration of life purpose. It is difficult to explain this in an exact manner because the mechanism by which gestalts — the frozen, painful now — are displaced from the whole to a part is not thoroughly understood by science. Indeed, the organization of the flow from the cells in the organism to the conscious mind is not thoroughly understood.

However, it would appear that cells merge their internal information systems and fields of consciousness when they join and form tissues and organs, which in turn merge into organ systems, which in turn are united in the body as a whole. The unity is not complete, however, since each level of the body and all its parts retain some degree of autonomy and a constant, active, and alert willingness to help. A major or minor part of the body can, therefore, spontaneously be assigned the function of accommodating intense emotional pain, the purpose being the survival of the organism as a whole. A good theory on exactly how this takes place has yet to be found. It is quite odd that our tissues and organs are independent and intelligent, cooperative and able to make autonomous decisions. In principle, it is no more odd than the assumption that the brain, which is also such an organ consisting of cells, is able to make decisions on its own, which very few people would doubt! The brain and the bowel, for example, are really not that different. Obviously, their decisions relate to different things, but they invariably serve the totality, its survival, and development in life.

When cells and tissues assume the task of accommodating a gestalt instead of attending to their original functions in the body, this will cause severe disruption. For example, in connection with the immune system, such blockages can provide the tissue with a status of nonself, which means that it falls victim to an autoimmune attack. When the cells are withdrawn from the totality, the control that normally prevents the cells from dividing uncontrollably disappears, leading to the development of the disease referred to as cancer. Depending on which cells are withdrawn from the organism and in what way, a wide range of different diseases occur. This theory would be strange, complex, and poor if it were not supported directly by experience.

Our experience with applied holistic medicine founded on the purpose of life now dates back several years. Apparently, patients who are willing and able to sort out their lives so thoroughly that they find themselves again, and can contain their life purpose and live accordingly, will recover almost irrespective of the nature of their disease. Unfortunately, we do not yet have enough examples to provide sufficiently strong evidence to support this hypothesis. We have only worked in depth with about 30 patients suffering from completely different diseases and ailments, so this field still requires a great deal of research, in terms of both quality and quantity. Having said that, any person who breaks through to his or her life purpose and is able to live accordingly will, in our clinical experience, also recover, regardless of the nature of the particular disease — even in the case of very serious and potentially life-threatening diseases[31,32]. When we have worked with the patients according to the purpose of life theory and have made them break through and acknowledge their life purpose, very often great — and in rare cases almost miraculous — things have happened to the patients with very different diagnoses such as chronic pain, tinnitus, cancer, and sclerosis. Scientifically, it may appear surprising that a psychosomatic approach is effective in virtually any disease, but that appears to be the case. Thus, the door has been opened to a new era of psychosomatic medicine of the type that we refer to as holistic medicine or, to be more precise, consciousness-based medicine.

## CONCLUSION

Sometimes the patient has many different diseases, both somatic and mental. Biomedicine will normally see and treat each disease individually and, with many diseases and effects of the many drugs used, that can become a burden in itself, preventing the patient from getting better. With holistic medicine, all the diseases are seen as whole, as symptoms of a more fundamental imbalance in the state of being. What becomes the focus of the holistic treatment is the intention to understand the cause of all the different diseases and, thus, to be able to cure the patient. The disease is caused by the patient’s poor quality of life and the fundamental existential imbalances.

The holistic physician must help the patient to recover his or her existence and create a good relationship with self. The life mission theory, the theory of character, and the holistic process theory of healing stress that recovering the purpose of life (the life mission) and the physical, mental, and spiritual character is what is needed for the patient to regain life, love, and trust in order to find happiness and realize the true purpose of life.

We illustrate the power of holistic medicine with a case study, an invalidated female artist, aged 42 years, who suffered from multiple health problems, many of which had been chronic for years: tinnitus, 14-days menstruation in each cycle, migraine with severe scotomata blinding her for up to 20 min, often herpes simplex 1 and 2 on lip and labia, neck tensions, phlegm in the throat, tendency to cry, inferiority feeling, fungal infection in the crotch, hemorrhoids, severe abdominal complaints, mild depression, occasional minor hallucinations in the form of symbols, signs and aura vision which she could not control, feelings of desolation, and anxiety. After the treatment, her tinnitus disappeared and she was able to work again. The treatment was a combined strategy of improving the quality of life, recovering her human character and purpose of life ("renewing the patients life energy", "balancing her global information system"), and processing the local blockages, thus curing most of her many different diseases in one treatment using 30 h of intense holistic therapy over a period of 18 months.

## ACKNOWLEDGMENTS

This study was supported by grants from IMK Almene Fond. The quality of life research was approved by the Copenhagen Scientific Ethical Committee under number (KF)V.100.2123/91.

## BIOSKETCHES

**Søren Ventegodt, MD,** is the director of the Quality of Life Research Center in Copenhagen, Denmark. He is also responsible for a Research Clinic for Holistic Medicine in Copenhagen and is a popular speaker throughout Scandinavia. He has published numerous scientific or popular articles and a number of books on holistic medicine, quality of life, and quality of working life. His most important scientific contributions are the comprehensive SEQOL questionnaire, the very short QoL5 questionnaire, the integrated QOL theory, the holistic process theory, the life mission theory, and the ongoing Danish Quality of Life Research Survey, 1991–94 in cooperation with the University Hospital of Copenhagen and the late professor of pediatrics, Bengt Zachau-Christiansen, MD, PhD. E-mail: ventegodt@livskvalitet.org. Website: http://www.livskvalitet.org

**Joav Merrick, MD, DMSc,** is professor of child health and human development affiliated with the Zusman Child Development Center, Division of Pediatrics and Community Health at the Ben Gurion

University, Beer-Sheva, Israel; the medical director of the Division for Mental Retardation, Ministry of Social Affairs, Jerusalem; and founder and the director of the National Institute of Child Health and Human Development. He has numerous publications in the field of child health and human development, rehabilitation, intellectual disability, disability, health, welfare, abuse, advocacy, quality of life, and prevention. Dr. Merrick received the Peter Sabroe Child Award for outstanding work on behalf of Danish Children in 1985 and the International LEGO-Prize ("The Children's Nobel Prize") for an extraordinary contribution towards improvement in child welfare and well being in 1987. E-mail: jmerrick@internet-zahav.net. Website: www.nichd-israel.com
